# Is It Time to Test the Antiseizure Potential of Palmitoylethanolamide in Human Studies? A Systematic Review of Preclinical Evidence

**DOI:** 10.3390/brainsci12010101

**Published:** 2022-01-12

**Authors:** Riccardo Bortoletto, Matteo Balestrieri, Sagnik Bhattacharyya, Marco Colizzi

**Affiliations:** 1 Child and Adolescent Neuropsychiatry Unit, Maternal-Child Integrated Care Department, Integrated University Hospital of Verona, 37126 Verona, Italy; riccardo.bortoletto@studenti.univr.it; 2 Unit of Psychiatry, Department of Medicine (DAME), University of Udine, 33100 Udine, Italy; matteo.balestrieri@uniud.it; 3 Department of Psychosis Studies, Institute of Psychiatry, Psychology and Neuroscience, King’s College London, London SE5 8AF, UK; sagnik.2.bhattacharyya@kcl.ac.uk

**Keywords:** seizure, convulsion, cannabinoids, acylethanolamines, immune response, glutamate, inflammation, peroxisome proliferator-activated receptor-α, neurology

## Abstract

Antiseizure medications are the cornerstone pharmacotherapy for epilepsy. They are not devoid of side effects. In search for better-tolerated antiseizure agents, cannabinoid compounds and other N-acylethanolamines not directly binding cannabinoid receptors have drawn significant attention. Among these, palmitoylethanolamide (PEA) has shown neuroprotective, anti-inflammatory, and analgesic properties. All studies examining PEA’s role in epilepsy and acute seizures were systematically reviewed. Preclinical studies indicated a systematically reduced PEA tone accompanied by alterations of endocannabinoid levels. PEA supplementation reduced seizure frequency and severity in animal models of epilepsy and acute seizures, in some cases, similarly to available antiseizure medications but with a better safety profile. The peripheral-brain immune system seemed to be more effectively modulated by subchronic pretreatment with PEA, with positive consequences in terms of better responding to subsequent epileptogenic insults. PEA treatment restored the endocannabinoid level changes that occur in a seizure episode, with potential preventive implications in terms of neural damage. Neurobiological mechanisms for PEA antiseizure effect seemed to include the activation of the endocannabinoid system and the modulation of neuroinflammation and excitotoxicity. Although no human study was identified, there is ground for testing the antiseizure potential of PEA and its safety profile in human studies of epilepsy.

## 1. Introduction

According to the International League Against Epilepsy (ILAE), seizures can be described as the “transient occurrence of signs and/or symptoms due to abnormal excessive or synchronous neuronal activity in the brain.” Epilepsy subsists when the patient’s brain shows an augmented tendency to the recurrence of seizures [1]. It is one of the most frequent neurological disorders, involving about 50 million people worldwide, mainly in developing countries. It shows a U-shaped distribution in terms of age, with a first peak during middle childhood (5–9 years) and a second one around 80 years of age, affecting all races and both genders [2]. While childhood-onset epilepsy is more likely to be idiopathic, late-onset epilepsy is likely to be due to an identifiable cause, including trauma, central nervous system (CNS) infections, space-occupying lesions, cerebrovascular accidents (CVA), metabolic disorders, and drugs [3]. Chronic use of antiseizure medications (ASMs) is still considered as the cornerstone pharmacotherapy for seizures even though it might expose the patient to major somatic adverse effects, such as fatigue, dizziness, sedation, headache, and nausea, as well as neuropsychiatric problems, such as anxiety, depression, or sleep disorders, especially when polytherapy is needed [4]. Benzodiazepine drugs (BDZ) represent a frequently used therapeutic option: seizures are often efficiently reduced in the early stages of treatment through the production of allosteric changes in GABA-A receptors, which lead to an increase of GABAergic neurotransmission and to a decreased neuronal excitability. BDZs are not devoid of causing unpleasant drowsiness and incoordination and may affect cognitive performance alongside the eventual development of physical dependence and tolerance [5].

The search is on for novel antiseizure agents with fewer adverse effects. Cannabinoid compounds as ASMs via the cannabinoid receptor type 1 (CB1) have already shown promising results [6,7]. The endocannabinoids (eCBs) anandamide (AEA) and 2-arachidonoyl-glycerol (2-AG), as well as the N-acylethanolamines palmitoylethanolamide (PEA) and N-oleylethanolamine (OEA), which act as endogenous lipid signaling molecule analogues not directly binding CB1 receptor, are under investigation as possible therapeutic options for CNS diseases, including the control of epileptic seizures. PEA has firstly been identified in egg yolk, soy bean, and peanut oil and subsequently detected in mammalian tissues. It is an endogenous fatty acid amide, which exerts its biological effects through the activation of peroxisome proliferator-activated receptor-α (PPAR-α) and its related independent pathways, including ion channels involved in neuronal firing and the Transient Receptor Potential Vanilloid 1 (TRPV1) receptor [8], whose role is considered crucial in the fulfilment of neuroprotective, anti-inflammatory, and analgesic properties [9]. PEA has been suggested as an effective treatment for inflammatory disorders and pain [10,11], together with possible therapeutic implications in depressive symptoms and autism spectrum disorder [12,13].

### Objectives

PEA effects on glutamate signaling, ion channels and systemic inflammation, and/or peripheral immunity activation could represent promising mechanisms to reduce the likelihood for seizures’ occurrence and progression. This review aimed to bring together and discuss all available data generated by studies investigating the role of PEA in epilepsy by conducting a systematic literature search for all such data. All interventional and observational studies have been reviewed.

## 2. Experimental Procedures

### 2.1. Inclusion and Exclusion Criteria

In order to summarize previous research on the subject, inclusion criteria for studies were as follows: (1) human or animal studies; (2) studies assessing the effects of PEA administration in epilepsy and acute seizures; and (3) studies investigating PEA signaling-related molecular markers in epilepsy and acute seizures, including (a) blood serum levels, (b) brain tissue levels, (c) peripheral tissue levels, (d) enzyme activity, and (e) receptors. Exclusion criteria were (1) studies where PEA was not the intervention considered (studies evaluating only exogenous cannabinoid agonists or antagonists or any eCBs other than PEA), (2) studies where PEA’s neuroprotective role was not evaluated with reference to epilepsy or acute seizures, and (3) studies in which PEA effects were not directly reported on.

### 2.2. Search Strategy and Data Extraction

A literature search was conducted using electronic databases (PubMed, Web of Science and Scopus) for any published original study written in English, using a combination of search terms describing and/or concerning epilepsy (‘epilep*’, ‘seizure’, ‘convuls*’, ‘tonic’, ’clonic’, ‘myoclon*’, ‘attack’, ‘paroxysm’, ‘tremor’ and ‘antiepileptic’) and PEA (‘palmitoylethanolamide’, ‘palmitylethanolamide’, ‘(N-(2-hydroxyethyl)hexadecanamide)’, ‘(N-(2-hydroxyethyl)palmitate)’, ‘N-palmitoylethanolamine’), on 4 December 2021. No predefined duration of PEA exposure, default gender, stage of life/epilepsy, or therapeutic strategies for study search was adopted in order to be the most inclusive as possible. Reference lists of eligible studies were screened to identify additional eligible research. Publication data screening and extraction were performed following a 2-step selection process (conventional double-screening) conducted by 2 reviewers independently of each other (R.B. and M.C.).

### 2.3. Risk of Bias

The methodological heterogeneity of the studies (Table 1) included in this review necessitated a suitably inclusive and flexible approach to assess risk of bias and study quality. An adapted set of criteria suggested by the Agency for Healthcare Research and Quality (AHRQ) guidance was used [14], amended as appropriate for interventional and observational studies in animals. To assess any factor that may account for similarities and differences between animal studies, information was extracted about study characteristics, including animal model (mouse or rat), seizure model (chemical stimulation, electrical stimulation, strain of idiopathic epilepsy), developmental stage (prenatal, postnatal, adult), gender, and PEA dosage and exposure (Table 2).

## 3. Results

### 3.1. Study Selection

**Figure 1:**
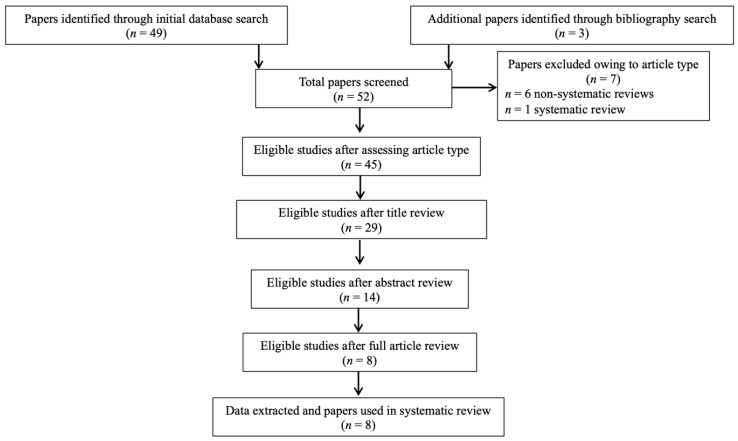
PRISMA flowchart of search strategy for systematic review.

In summary, 52 records were retrieved. After excluding articles owing to article type (systematic and non-systematic reviews), by using a three-step screening approach, titles, abstracts, or full texts of all records were screened against the inclusion and exclusion criteria (Figure 1). A final list of eight studies was used for systematic analysis in this review (Table 1). No eligible human study was identified. In total, the eligible studies assessed different aspects of the palmitoylethanolamide (PEA) signaling pathway (Table 1). These included (1) in-vivo single/acute PEA exposure in animal models of epilepsy (two studies) and acute seizures (three studies); (2) in-vivo acute vs. subchronic PEA exposure in animal models of acute seizures (one study); (3) in-vivo PEA vs. other eCBs/antiseizure medications (ASMs) exposure in animal models of acute seizures (one study); (4) PEA quantitative brain assessment in animal models of epilepsy (one study) and acute seizures (one study); (5) PEA quantitative brain assessment in young vs. adult animal models of acute seizures (one study); (6) PEA quantitative peripheral tissues assessment in animal models of acute seizures (one study); (7) eCB and eicosanoid (eiC) quantitative brain assessment in PEA-treated animal models of acute seizures (one study); and (8) eCB and eiC quantitative blood assessment in PEA-treated animal models of acute seizures (one study). Additional data on methodological quality of studies are reported in Table 2. A brief synthesis of the main results is presented below and summarized in Table 1.

### 3.2. In Vivo Acute and Subchronic PEA Treatment Exposure and Comparison with Other Endocannabinoids (eCBs) and ASMs Exposure in Animal Models of Epilepsy and Acute Seizures

Most studies identified in this review addressed the effects of PEA exposure in animal models of epilepsy and acute seizures using similar but not overlapping methodologies in terms of animal type (mice [15,16,17], rat [18,19,20]), mode of administration (intraperitoneal [15,16,17,18,19], intracerebroventricular [19,20]), period of exposure (from 14 days old to month 7 months old), dosage of PEA (0.5 to 250 mg/kg for intraperitoneal administration [15,16,18,19]; 0.5 to 10 µg/2 µL [19] or 1 to 25 µg/kg [20] for intracerebroventricular administration), and model of pathology (chemical stimulation [15,17,20], electrical stimulation [15,18], genetic models [16,19]). The first of these studies estimated the superiority of PEA to placebo, indicating antiseizure properties of the compound, with an effective dose (ED_50_) comparable to that of the ASM phenytoin (PHT) and a higher protective index [15]. While being effective in controlling tonic seizures, PEA was not effective against clonic convulsions, where it performed worse than PHT [15]. Except for some modest activity of anandamide (AEA) and palmitamide (PAA), other compounds related to eCBs or to the palmitic acid structure were devoid of ASM activity in the experimental conditions [15]. A subsequent study confirmed and extended such findings, supporting the evidence that PEA suppresses the tonic component in animal models of tonic-clonic seizure, increasing latency to clonus, and prolonging the latency between convulsive episodes [18]. Another study found that PEA administration reduces the epileptic spike-wave discharges (SWDs) in a widely validated genetic animal model for generalized absence epilepsy, the Wistar Albino Glaxo from Rijswijk (WAG/Rij) rat [19]. Such antiseizure effect was completely blocked by pretreatment with a synthetic cannabinoid receptor type 1 (CB1) antagonist/inverse agonist and a nuclear peroxisome proliferator-activated receptors (PPAR-α) antagonist [19]. While the synthetic CB1 antagonist/inverse agonist had pro-epileptic effects and interfered with the antiseizure activity of AEA, the PPAR-α antagonist did not have any effect and did not modify the antiseizure properties of AEA [19]. Studies carried out in a genetic model of reflex audiogenic epilepsy, the DBA/2 mouse [16], as well as by inducing seizures with a chemical kindling process [20] confirmed that the antiseizure effect of PEA is diminished by antagonizing CB1 and CB2 receptors [16,20] and PPAR-α [16], while the activation on the eCB system in the brain has ASM effects [16]. Co-administration of both PEA and CB1 and CB2 receptor agonists potentiated ASMs’ activity via pharmacodynamic mechanisms [16]. A more recent study was novel in indicating that the subchronic administration of PEA (double exposure at 7-h and 30-min prior to chemically induce the seizure) exerts larger beneficial effects as compared to single PEA injection (single exposure 30-min prior to chemically induce the seizure) in terms of attenuating both the behavioral (seizure intensity) and neurobiological (peripheral and hippocampal inflammatory responses to the excitotoxicity) seizure-related alterations [17]. Elevating PEA levels by inhibiting the fatty acid amide hydrolase (FAAH), which degrades it, resulted in similar effects than exogenously administering PEA [17]. Combinatorial administration of FAAH inhibitor with PEA did not produce any cumulative therapeutic effect in seizure alleviation [17]. Systemic pharmacological blockade of FAAH (occurring in the brain) rather than peripheral appeared to be required to successfully modulate inflammation and exert efficient antiseizure properties [17].

### 3.3. PEA Quantitative Brain and Peripheral Tissue Assessment and Comparison as a Function of Age in Animal Models of Epilepsy and Acute Seizures

In total, three studies did not evaluate the direct effect of PEA exposure while analyzing PEA levels in the brain and peripheral tissues of animal models of epilepsy. A study found that PEA levels in the amygdala, cortex, and thalamus are reduced in the genetic animal model of epilepsy, WAG/Rij, as compared to control animals, possibly compensated by an increase of cortical PEA levels at a later age, with such early and persistent decrease in PEA tone being accompanied by alterations in the eCB levels [19]. Similar results were found in another report, indicating lower PEA levels in the striatum and cerebellum but not in cerebral cortex, thalamus, hypothalamus, and hippocampus of animals presenting with chemically induced epilepsy versus controls [21]. This study identified reduced PEA levels in the lung and plasma in the context of epilepsy. Brain region- and periphery tissue-specific alterations were observed for eCBs, acylethanolamines, phospholipids, and eiCs [21]. A study identified differential responses as a function of age in an animal model of epilepsy, indicating higher hippocampal PEA levels in young animals but lower levels in the same region in adult animals, with differential responses in terms of eCB levels [22].

### 3.4. Endocannabinoid (eCB) and Eicosanoid (eiC) Quantitative Brain and Blood Assessment in PEA-Treated Animal Models of Acute Seizures

This systematic review identified a single study specifically investigating whether epileptic animals differ in terms of brain and blood levels of eCB and eiC levels as a function of PEA treatment. This study found that the acute seizure phase represents a turn-over time point in the dynamic of eCB and eiC level changes. Specifically, they expressed an increase in hippocampus accompanied by a decrease in periphery before the acute phase and normalization to basal hippocampal levels followed by augmentation in periphery after the acute phase [17]. PEA administration completely restored to basal such hippocampal increase, which occurs in early response to the chemically induced excitotoxicity, with subsequent reduction of seizure intensity. Similar normalizing effects of PEA were observed in periphery, supporting the notion of a modulation of eCB and eiC levels across the periphery-brain axis [17].

## 4. Discussion

This is the first systematic review of all studies investigating the behavioral effects of palmitoylethanolamide (PEA) and their neurobiological underpinnings in seizures and epilepsy. All records identified consisted of animal studies, while no research conducted in humans was available. Previous reviews had mainly focused on the role of major phytocannabinoids, indicating that they may represent a complementary tool for the symptomatic management of refractory epilepsy [23]. Specifically, research findings converged on the efficacy of pure cannabidiol (CBD), the isolated chemical product, and CBD-enriched cannabis extracts, where the therapeutic effect is supposedly driven by the complex interactions between all the components of the cannabis plant and remains poorly understood [24]. Such evidence led to Epidiolex, a cannabis plant-derived oral CBD solution, becoming licensed in the United States and Europe for treatment-resistant severe forms of childhood epilepsy, such as Lennox–Gastaut syndrome and Dravet syndrome [25]. Research studies on its therapeutic properties indicate that CBD is a negative allosteric modulator of cannabinoid (CB) receptors [26] and may modulate endogenous endocannabinoid (eCB) levels by indirect mechanisms involving the nuclear receptor peroxisome proliferator-activated receptor-α (PPAR-α) and PPAR-α-independent pathways, such as Transient Receptor Potential Vanilloid 1 (TRPV1) and G55 protein-coupled receptor (GPR55) [27]. Consistent with this, recent research highlighted the importance of widening the investigation to cannabinoid-related compounds whose actions depend on the interaction with non-CB receptors [28]. Overall, this review demonstrated that PEA, a naturally occurring N-acylethanolamine whose biological effects are related to indirect activation of CB1 receptors as well as PPAR-α and TRPV1 modulation [8,19], may be involved in seizures and epilepsy. Evidence was obtained from interventional studies of the positive behavioral and neurobiological effects of PEA supplementation, observational studies of aberrancies in the PEA tone, and studies of PEA-mediated modulation of eCB levels across the periphery-brain axis in the context of seizures and epilepsy.

PEA supplementation in animal models of epilepsy and acute seizures was found to reduce seizure frequency and severity [15,16,17,18,19,20], in some cases, similarly to available ASMs but with a better safety profile in terms of a higher therapeutic index as a ratio between the amount of drug that causes toxicity and the amount that causes the therapeutic effect [15]. Potential neurobiological mechanisms for such antiseizure effect included the activation of the eCB system [16,19,20] and the modulation of neuroinflammation and excitotoxicity [17]. In particular, eCB system activation has been suggested to exert neuroprotective effects by modulating glutamate neurotransmission [29,30,31,32] and GABAergic tone [33,34,35,36]. The peripheral-brain immune system appeared to be more effectively modulated by subchronic pretreatment with PEA [17], possibly because of a resulting state of ‘‘hypervigilance’’ capable of responding more adequately to subsequent negative stimuli, such as the epileptogenic insult [37,38]. PEA treatment was found to restore the eCB and eicosanoid (eiC) level changes that occur in acute and post-acute phases of an epileptic episode, with potential preventive implications in terms of neural damage [17].

Another line of research identified lower brain levels of PEA and related alterations in the eCB levels in animal models of epilepsy and acute seizures [19,21], dependent on the disease progression [22]. Brain PEA levels were found to be reduced only in adult epileptic animals while paradoxically increased in young animals [22]. PEA levels also appeared to be systemically reduced, as indicated by the finding of lower levels in the lung and plasma of epileptic animals [21].

The findings of this systematic review must be seen considering some limitations. Research in the field is still in its infancy, and animal experiments often do not translate into replications in human trials [39]. In the absence of human studies, no conclusions can be drawn about the relevance of PEA for the different clinical phenotypes of epilepsy. While limited evidence supports a neuroprotective effect of PEA against neuroinflammation and glutamate toxicity in the context of different neuropsychiatric conditions [13], studies are needed to elucidate the exact mechanism of action of PEA in epilepsy. Caution is needed about the finding of reduced PEA tone in epilepsy. The limited available evidence calls for research about the potential role of PEA concentration profile as a biomarker for the prevention, assessment, and management of epilepsy. Apart from a single observational study conducted in animals of different age [22] and a single study of subchronic pretreatment with PEA [17], no longitudinal evidence was available about the longer-term clinical utility of such a treatment. Except for one study, all evidence was gathered from male animal models, and no data were available on gender-dependent PEA effect in epilepsy [22]. Single evidence that PEA co-administration potentiates ASMs’ activity via pharmacodynamic mechanisms [16] requires replication. Clinical trials are needed to fully explore the efficacy and tolerability of PEA supplementation in epilepsy.

## 5. Conclusions

This review found a paucity of observational and experimental studies of PEA signaling in epilepsy. The eight investigations presented seemed to converge on the presence of PEA aberrancies in the brain and periphery, their role for the manifestations of different forms of seizures and epilepsy, and beneficial effects of PEA supplementation both in terms of antiseizure and antiepileptic effect. Subchronic pretreatment with PEA before the epileptogenic stimulus appeared to be a strategy to prevent its behavioral and neurobiological consequences, warranting investigation of PEA disease-modifying effect in epilepsy. It is time to test the antiseizure potential of PEA and its safety profile in human studies of epilepsy.

## Figures and Tables

**Table 1:** Summary of studies investigating palmitoylethanolamide and its correlations to epilepsy and acute seizures.

Study (Country)	Aim of Study	PEA Type of Study	Population	N	Outcome Measure (Test Name or Description)	Seizure Model	Summary Results
Lambert et al. (2001) (Belgium)	1. To assess PEA effects in tonic-clonic seizures in mice; 2. To compare PEA effects to other ECBs/palmitoyl derivatives and ASMs	In-vivo exposure in animals	1. MES test, 16 groups: (a) VHI (30 min/4 h); (b) PEA (100 mg/kg) (30 min/4 h); (c) PEA (50 mg/kg) (30 min/4 h); (d) AEA (30 min/4 h); (e) PA (30 min/4 h); (f) PAA (30 min/4 h); (g) HXD (30 min/4 h); (h) 16HPA (30 min/4 h); 2. Time course calculation, 8 groups: (a) 30 min; (b) 1 h; (c) 1 h 30 min; (d) 2 h; (e) 3 h; (f) 4 h; (g) 5 h; (h) 7 h; 3. ED50 calculation, 8 groups: (PEA range 0.5–50 mg/kg); 4. CIS test, 18 groups: (a) PTZ (VHI, PEA, PHT); (b) MPA (VHI, PEA, PHT); (c) BC (VHI, PEA, PHT); (d) STR (VHI, PEA, PHT); (e) PIC (VHI, PEA, PHT); (f) NMA (VHI, PEA, PHT); 5. Rotarod test group	1. MES test: 15–16 per group; 2. Time course calculation: 7–8 per group; 3. ED50 calculation: 6–10 per group; 4. CIS test: 15–16 per group; 5. Rotarod test: 6–10 per group	1. Antiseizure activity (MES test, CIS test); 2. Neurologic impairment (rotarod performance test)	1. Maximal electroshock seizure; 2. Chemically-induced seizure	ED50 was comparable between PEA and PHT; PEA was effective only against tonic seizures and showed a high protective index
Sheerin et al. (2004) (Canada)	To assess PEA effects in tonic-clonic seizures in rats	In-vivo exposure in animals	1. KS groups: 10 VHI (DMSO); 10 PEA (1); 8 PEA (10); 4 PEA (100); 2. CIS groups: 5 VHI; 5 PEA	42	Antiseizure activity (KS test; CIS test)	1. Kindled amygdaloid seizure; 2. PTZ-induced seizure	PEA increased latency to clonus at 1 mg/kg, was effective against tonic seizures, and increased the latency between convulsive episodes
Citraro et al. (2013) (Italy)	1. To assess PEA effects and PPAR-α role in absence seizures in rats; 2. To quantify PEA and other ECBs/AEs brain levels in epileptic and control rats	1. In-vivo exposure in animals; 2. Quantitative brain assessment in animals	1. 6 VHI (icv); 2. 6 VHI (ip); 3. 24 PEA (icv); 4. 24 PEA (ip); 5. 24 AEA (icv); 6. 18 SR (icv); 7. 12 GW (icv); 8. 12 SR (icv) + PEA(ip); 9. 6 GW(icv) + PEA (icv); 10. 6 SR (icv) + AEA (ip); 11. 6 GW (icv) + AEA (icv)	144	1. Antiepileptic activity (EEG recording); 2. Brain ECBs/AEs levels (LC/APCI/MS)	Genetic model of absence epilepsy	PEA showed anti-absence properties depending on PPAR-α and indirect CB1 receptors activation
Fezza et al. (2014) (Italy)	To quantify PEA and other ECBs/AEs brain levels in epileptic and control young and adult rats	Quantitative brain assessment in animals	1. In-vivo saline-injection: (a) 5 P14; 11 P56–70; 2. In-vivo KA-injection: (a) 5 P14; (b) 12 P56–70; 3. In-vitro KA bath perfusion: (a) 27 P14; (b) 36 P56–70	96	1. Brain ECB system activity (TLC, LC-ESI-MS); 2. Epileptic activity post ECB system manipulation (burst duration, burst amplitude, PSs number)	KA-induced seizure	PEA levels were higher in the hippocampus of younger KA-treated rats while decreasing in adults
Aghaei et al. (2015) (Iran)	To assess PEA effects in tonic-clonic seizures in rats	In-vivo exposure in animals	1. PTZ; 2. VHI (PTZ + saline or DMSO); 3. PEA + PTZ; 4. AM630 + PTZ; 5. AM630 + PEA + PTZ; 6. AM251 + PTZ; 7. AM251 + PEA + PTZ; 8. AM251 + AM630 + PTZ; 9. AM251 + AM630 + PEA + PTZ	220	Antiseizure activity (CIS test)	PTZ-induced seizure	PEA reduced PTZ-induced seizures, and CB1/CB2 blockage reduced its effectiveness
Citraro et al. (2016) (Italy)	To assess PEA effects in tonic-clonic seizures in mice	In-vivo exposure in animals	1. VHI; 2. PEA, 3. GW + PEA, 4. NIDA + PEA; 5. ACEA, 6. GW + ACEA, 7. NIDA + ACEA; 8. WIN, 9. GW + WIN, 10. NIDA + WIN; 11. (ASMs) + VHI/PEA/WIN/ACEA/NIDA/GW	X	1. Antiepileptic activity (audiogenic seizures test); 2. Neurologic impairment (rotarod performance test); 3. Brain/plasma ASMs levels	Genetic model of reflex audiogenic epilepsy	PEA showed antiepileptic properties and potentiated the effect of several ASMs
Lerner et al. (2017) (Germany)	To quantify PEA, other ECBs/AEs and PLs/eiCs brain and peripheral tissue levels in epileptic and control mice	Brain and other tissues assessment in animals	1. 9 KA; 2. 9 saline	18	Lipid profiling (LC/MRM quantitative and qualitative assessment of PLs/ECBs/AEs/eiCs)	KA-induced seizure	PEA levels were lower in the striatum, cerebellum, lung, and plasma of epileptic animals compared to controls
Post et al. (2018) (Germany)	1. To assess acute and subchronic PEA effects in tonic-clonic seizures in mice; 2. To assess PEA effects on ECBs/eiCs brain and plasma levels; 3. To assess PEA effects on neurodegeneration	1. In-vivo exposure in animals; 2. Quantitative brain and blood assessment in animals	1. 48 KA; 2. 24 PEA2/KA; 3. 48 PEA/KA; 4. 24 CTRL1; 5. 24 CTRL2; 6. 24 PEA2; 7. 24 PEA + URB597/KA; 8. 24 URB597/KA; 9. 24 PEA + URB937/KA; 10. 24 URB937/KA (*n* = 6 per time point each group)	288	1. Antiepileptogenic activity (behavioral assessment in KA-induced seizures, LC/MRM quantification of ECBs/eiCs); 2. Hippocampus neurodegenerative process (immunohistochemistry)	KA-induced seizure	PEA subchronic administration reduced seizure intensity, enhanced neuroprotection, and modulated ECBs/eiCs brain and plasma levels
**Study (Country)**	**Results**						
Lambert et al. (2001) (Belgium)	1. MES test: **(a) % mice exhibiting seizures after 30 min: PEA (100–50 mg/kg), AEA, PAA < VHI**; PA, HXD, 16HPA vs. VHI, NS; (b) Time of peak effect, ED50: PEA vs. PHT, NS; 2. CIS test: **(a) % mice exhibiting clonic seizures**: PEA vs. VHI, NS after each compound; **PEA > PHT after PTZ, BC; (b) % mice exhibiting tonic seizures: PEA < VHI after PTZ, MPA, BC**; PEA vs. VHI, NS after STR, PIC, NMA; PEA vs. PHT, NS after PTZ, MPA, BC; **PEA > PHT, after PIC;** 3. Rotarod test: **(a) TD50: PEA > Ameltolide, PHT, CBZ, PB; PEA < VPA; (b) PI (TD50/ED50): PEA > each ASM**						
Sheerin et al. (2004) (Canada)	1. KS test: **(a) latency to clonus: PEA(1)**, ↑; VHI, PEA(10), PEA(100), NS; (b) Duration of clonus: VHI, PEA(1), PEA(10), PEA(100), NS; (c) AD duration: VHI, PEA(1), PEA(10), PEA(100), NS; 2. CIS test: **(a) tonic seizures: PEA < VHI**; (b) clonic convulsions: PEA vs. VHI, NS; (c) 1st convulsion latency, PEA vs. VHI, NS; (d) 1st convulsion duration, PEA vs. VHI, NS; (e) **2nd convulsion latency, PEA > VHI**; (f) 2nd convulsion duration, PEA vs. VHI, NS						
Citraro et al. (2013) (Italy)	1. **SWDs number and duration: PEA (1, 3, and 10 µg/2 µL (icv) and 20, 40, and 60 mg/kg (ip)) < VHI**; PEA (0.5 µg/2 µL (icv) and 10 mg/kg (ip)) vs. VHI, NS; **AEA < VHI (1, 3, and 10 µg/2 µL) with dose-dependent effect**; AEA vs. VHI (0.5 µg/2 µL), NS; **SR > VHI**; GW vs. VHI, NS; SR (icv) + PEA (ip) vs. VHI, NS; GW (icv) + PEA (icv) vs. VHI, NS; SR (icv) + AEA (ip) vs. VHI, NS; **GW (icv) + AEA (icv) < VHI;** 2. **AEA levels: ↓ with age in WAG/Rij in amygdala and cortex; ↓ with age in Wistar in thalamus; 2-AG levels: ↑ with age in ACI in amygdala; ↑ with age in Wistar in cortex; PEA levels: ↓ with age in ACI in thalamus; amygdala PEA levels: 6-month WAG-Rij < 6-month Wistar, ACI; cortex PEA levels: 6-month WAG-Rij > 6-month Wistar, ACI; thalamus PEA-levels: 2/6-month WAG-Rij < 2/6-month Wistar, ACI; amygdala AEA and 2-AG levels: 6-month WAG/Rij < 6-month ACI**; 6-month WAG/Rij vs. 6-month Wistar, NS.						
Fezza et al. (2014) (Italy)	1. Hippocampi ECB system analysis post saline: **PEA and AEA levels, FAAH and NAPE-PLD activity, CBR binding affinity: P14 < P56–70**; MAGL and DAGL activity, 2-AG levels: P14 vs. P56–70, NS; **2. Seizure onset rapidity post KA: P14 > P56–70**; female P56–70 vs. male P56–70, NS; 3. Hippocampi ECB system analysis post KA: FAAH and MAGL activity, CBR binding affinity: KA/P14 vs. saline/P14, KA/P56–70 vs. saline/P56–70, NS; **PEA and AEA levels, NAPE-PLD activity: KA/P14 > saline/P14, KA/P56–70 < saline/P56–70; 2-AG levels, DAGL activity: KA/P14 < saline/P14, KA/P56–70 > saline/P56–70;** **4. Burst duration: KA > saline; P14 > P56–70;** P14: KA vs. URB597 + KA, NS; **P14: KA > JZL + KA; P56–70: KA > URB597 + KA;** P56–70: KA vs. JZL + KA, NS; **P56–70: KA > KA + WIN; P56–70: KA + WIN < KA + WIN + SR; P56–70: KA < SR; KA < KA + SR**; 5. **Burst amplitude: KA > saline; P14 > P56–70; P14: KA > URB597 + KA; P14: KA > KA + WIN; P56–70: KA + WIN < KA + WIN + SR**; P56–70: KA vs. SR, NS. **6. PSs number: P56–70: KA > KA + WIN**; P56–70: KA vs. SR, NS; **P56–70: KA < KA + SR.**						
Aghaei et al. (2015) (Iran)	**1. LP: PEA + PTZ > VHI**; AM251 + PTZ (1.25, 2.5, 5 µg/kg) vs. VHI, NS; **AM251 + PTZ (10 µg/kg) > VHI**; AM630 + PTZ (2.5, 5 µg/kg) vs. VHI, NS; **AM630 + PTZ (10, 20, 40 µg/kg) < VHI; PEA + PTZ > AM251 + PEA + PTZ > VHI; PEA + PTZ > AM630 + PEA + PTZ > VHI; AM251 + AM630 + PTZ < VHI; PEA + PTZ > AM251 + AM630 + PEA + PTZ > VHI**; **2. S5D: PEA + PTZ < VHI; AM251(1.25, 2.5 µg/kg) + PTZ > VHI; AM251 (5, 10 µg/kg) + PTZ < VHI**; AM630 (2.5 µg/kg) + PTZ vs.VHI, NS; **AM630 (5, 10, 20, 40 µg/kg) + PTZ > VHI; PEA + PTZ < AM251 + PEA + PTZ < VHI; AM251 + AM630 + PTZ > VHI; PEA + PTZ < AM251 + AM630 + PEA + PTZ < VHI; PEA + PTZ < AM630 + PEA + PTZ < VHI**; **3. S5L(-1): PEA + PTZ < VHI; AM251(5, 10 µg/kg) + PTZ < VHI; AM251 (2.5 µg/kg) + PTZ > VHI; **AM630 (2.5 µg/kg) + PTZ vs.VHI, NS; **AM630 (5, 10, 20, 40 µg/kg) + PTZ > VHI; PEA + PTZ < AM251 + PEA + PTZ < VHI; AM251 (2.5 µg/kg) + AM630 (20 µg/kg) + PTZ > VHI; VHI > AM251 + AM630 + PEA + PTZ > PEA + PTZ; PEA + PTZ < AM630 + PEA + PTZ < VHI**; **4. SS: PEA(2.5, 5, 10, 25 µg/kg) + PTZ < VHI**; PEA (1 µg/kg) + PTZ vs. VHI, NS; **AM251 (5, 10 µg/kg) + PTZ < VHI**; AM251 (1.25, 2.5 µg/kg) + PTZ vs. VHI, NS; AM630 + PTZ vs. VHI, NS						
Citraro et al. (2016) (Italy)	1. (a) **ED50, wild running phase: PEA (60 min) < GW (30 min) + PEA (60 min); ACEA (60 min) < NIDA (45 min) + ACEA (60 min); WIN (20 min) < NIDA (45 min) + WIN (20 min)**; differences among other concurrent groups, NS; **(CBZ, DZP, FBM, GPT, LTG, OXC, PB, TPM, VPA) + PEA < (same ASMs) + VHI**; (LEV, PHT) + PEA vs. (same ASMs) + VHI, NS; **(CBZ, DZP, FBM, GPT, LTG, TPM, VPA) + WIN/ACEA < (same ASMs) + VHI**; (LEV, PHT, OXC, PB) + WIN/ACEA vs. (same ASMs) + VHI, NS; ASMs + NIDA/GW vs. ASMs + VHI, NS; (b) **ED50, clonic phase: PEA (90 min) < NIDA (45 min) + PEA (90 min); PEA (60 min) < GW (30 min) + PEA (60 min); PEA (90 min) < GW (30 min) + PEA (90 min); WIN (20 min) < NIDA (45 min) + WIN (20 min); ACEA (60 min) < NIDA (45 min) + ACEA (60 min)**; differences among other concurrent groups, NS; **(CBZ, DZP, FBM, GPT, LTG, OXC, PB, TPM, VPA) + PEA < (same ASMs) + VHI**; (LEV, PHT) + PEA vs. (same ASMs) + VHI, NS; **(CBZ, DZP, FBM, GPT, LTG, PB, TPM, VPA) + WIN < (same ASMs) + VHI**; (LEV, PHT, OXC) + WIN vs. (same ASMs) + VHI, NS; **(CBZ, DZP, FBM, GPT, PB, VPA) + ACEA < (same ASMs) + VHI**; (LEV, PHT, OXC, LTG, TPM) + ACEA vs. (same ASMs) + VHI, NS; ASMs + NIDA/GW vs. ASMs + VHI, NS; (c) **ED50, tonic phase: PEA (60 min) < NIDA (45 min) + PEA (60 min); PEA (90 min) < NIDA (45 min) + PEA (90 min); PEA (60 min) < GW (30 min) + PEA (60 min); PEA (90 min) < GW (30 min) + PEA (90 min); WIN (20 min) < NIDA (45 min) + WIN (20 min); ACEA (60 min) < NIDA (45 min) + ACEA (60 min)**; differences among other concurrent groups, NS; **(CBZ, DZP, FBM, GPT, LTG, OXC, PB, TPM, VPA, PHT) + PEA < (same ASMs) + VHI**; LEV + PEA vs. LEV + VHI, NS; **(CBZ, DZP, FBM, GPT, LTG, PB, TPM, VPA) + WIN < (same ASMs) + VHI**; (LEV, PHT, OXC) + WIN vs. (same ASMs) + VHI, NS; **(CBZ, DZP, FBM, GPT, PB, VPA, TPM) + ACEA < (same ASMs) + VHI**; (LEV, PHT, OXC, LTG) + ACEA vs. (same ASMs) + VHI, NS; ASMs + NIDA/GW vs. ASMs + VHI, NS. 2. **TD50, (DZP, FBM, GPT, LTG, OXC, PB, TPM, VPA, PHT) + PEA < (same ASMs) + VHI**; (LEV, CBZ) + PEA vs. (same ASMs) + VHI, NS; **(DZP, FBM, GPT, LTG, OXC, PB, TPM, VPA) + WIN < (same ASMs) + VHI**; (LEV, CBZ, PHT) + WIN vs. (same ASMs) + VHI, NS; **(DZP, FBM, GPT, LTG, PB, PHT, TPM, VPA) + ACEA < (same ASMs) + VHI**; (LEV, CBZ, OXC) + ACEA vs. (same ASMs) + VHI, NS; ASMs + NIDA/GW vs. ASMs + VHI, NS. 3. Brain/plasma ASMs levels: ASMs + PEA/WIN/ACEA/NIDA vs. ASMs + VHI, NS.						
Lerner et al. (2017) (Germany)	(a) **cCTX levels: PA 16:0_18:1 and SM d18:1/18:0, KA > saline; PE 16:0_18:1, PE 20:2_20:4, 12(S)-HETE and 15(S)-HETE, KA < saline**; other PLs, ECBs, AEs and eiCs, KA vs. saline, NS; (b) **CER levels: OEA, PEA, LPC 20:4 and PC 18:2_20:4, KA < saline**; other PLs, ECBs, AEs and eiCs, KA vs. saline, NS; (c) **THL levels: PE 20:0_20:4 and C16:0, KA > saline; PA 16:0_18:1 and PS 16:0_18:1, KA < saline**; other PLS, ECBs, AEs and eiCs, KA vs. saline, NS; (d) HYP levels: PE 16:0_18:1, PG 18:1_20:4, PE 18:0_20:4, PE 20:2_20:4, LPC 18:0, PC 16:0_18:1, PG 16:0_18:1, PI 16:0_18:1, PC 18:2_20:4, PC 18:0_20:4, PG 16:1_20:4, PE 18:2_20:4, PE 20:0_20:4, PS 16:0_18:1, SM d18:1/18:0, LPA 16:0, LPA 20:4 and C18:1, KA < saline; PGD2, KA > saline; other PLS, ECBs, AEs and eiCs, KA vs. saline, NS; (e) **HC levels: PGD2 and PGF2α, KA > saline**; other PLS, ECBs, AEs and eiCs, KA vs. saline, NS; (f) **STR levels: PEA, PC 16:0_18:1 and LPA 20:4, KA < saline; PGF2α, KA > saline**; other PLS, ECBs, AEs and eiCs, KA vs. saline, NS; (g) **Heart levels: SM d18:1/18:0, PG 16:0_18:1, PS 16:0_18:1, PI 16:0_18:1, C18:1, C16:0 and C20:4, KA > saline; PC 18:0_20:4 and PC 18.2_20:4, KA < saline**; other PLS, ECBs, AEs and eiCs, KA vs. saline, NS; (h) **Lung levels: PEA and LPA 20:4, KA > saline; PC 16:0_18:1, LPA 16:0 and PG 16:0_18, KA < saline**; other PLS, ECBs, AEs and eiCs, KA vs. saline, NS; (i) **Plasma levels: AEA, OEA, PEA, AA, PG 16:0_18:1, PS 16:0_18:1, SM d18:1/18:0, PE 16:0_18:1 and PG 18:1_20:4, KA < saline; 2-AG and PGD2, KA > saline**; other PLS, ECBs, AEs and eiCs, KA vs. saline, NS.						
Post et al. (2018) (Germany)	1. (a) **Mean Behavioral Score: 20 min post KA: PEA/KA < KA**; PEA2/KA vs. KA, NS; **40, 60, 90, 120 min post KA: PEA/KA, PEA2/KA < KA; 150, 180 min post KA: PEA2/KA < KA**; PEA/KA vs. KA, NS; 180 min post KA: URB597/KA vs. PEA/KA vs. PEA + URB597/KA, NS; **10, 20 min post KA: PEA/KA < URB937/KA, PEA + URB937/KA; 40 min post KA: PEA/KA < PEA + URB937/KA; 60, 90 min post KA: PEA/KA, URB937/KA < PEA + URB937/KA; 120 min post: PEA/KA < PEA + URB937/KA**; URB937/KA vs. PEA + URB937/KA, NS; (b) **Hippocampus ECBs/eiCs levels (min post KA): AEA (20 min): KA > CTRL, PEA2/KA**; AEA (60, 120, 180 min): PEA2/KA vs. KA vs. CTRL, NS; 2-AG (20, 60, 120, 180 min): PEA2/KA vs. KA vs. CTRL, NS; **PEA (20 min), PEA2/KA > both; PEA (60 min): PEA2/KA > CTRL**; PEA2/KA vs. KA, NS; PEA (120, 180 min): PEA2/KA vs. KA vs. CTRL, NS; AA (20 min): KA > both; AA (60, 120, 180 min): PEA2/KA vs. KA vs. CTRL, NS; **PGE2 (20 min): KA > CTRL, PEA2/KA**; PGE2 (60, 120, 180 min): PEA2/KA vs. KA vs. CTRL, NS; **PGD2 (20, 60 min): KA > both**; PGD2 (120, 180 min); PEA2/KA vs. KA vs. CTRL, NS; **AEA (20, 60, 180 min): PEA/KA < URB597/KA, PEA + URB597/KA; AEA (120 min): PEA/KA < URB597/KA**; PEA/KA vs. PEA + URB597/KA, NS; 2-AG (20, 60, 120, 180 min): PEA/KA vs. URB597/KA vs. PEA + URB597/KA, NS; **PEA (20 min): PEA/KA < URB597/KA < PEA + URB597/KA; PEA (60, 120, 180 min); PEA/KA < both**; AA (20 min): PEA/KA vs. URB597/KA vs. PEA + URB597/KA, NS; **AA (60 min): PEA/KA < PEA + URB597/KA; AA(120 min): PEA/KA < URB597/KA; AA(180 min): PEA/KA < URB597/KA, PEA + URB597/KA**; PGE2 (20, 60 min): PEA/KA vs. URB597/KA vs. PEA + URB597/KA, NS; **PGE2 (120 min): PEA/KA < URB597/KA; PGE2 (180 min): PEA/KA < both; PGD2 (20 min): PEA/KA > PEA + URB597/KA**; PGD2 (60 min): PEA/KA vs. URB597/KA vs. PEA + URB597/KA, NS; **PGD2 (120 min): PEA/KA < URB597/KA; PGD2 (180 min): PEA/KA < both**; AEA, 2-AG, AA and PGD2 (20, 60, 120, 180 min): PEA/KA vs. URB937/KA vs. PEA + URB937/KA, NS; **PEA (20, 60, 120, 180 min): PEA/KA < URB937/KA, PEA + URB937/KA; PEA (20, 60, 120 min): URB937/KA < PEA + URB937/KA**; PGE2 (20, 60, 120 min): PEA/KA vs. both, NS. **PGE2 (180 min): PEA/KA < both**; (c) **Plasma ECBs/eiCs levels (min post KA): AEA (20, 60 min): CTRL > both; AEA (180 min); KA > both**; AEA (120 min): PEA2/KA vs. KA vs. CTRL, NS; **2-AG (60 min): KA > CTRL, PEA2/KA; 2-AG (180 min): PEA2/KA < CTRL**; 2-AG (20, 120 min): PEA2/KA vs. KA vs. CTRL, NS; **PEA (20, 60, 120, 180 min): PEA2/KA > both; AA (20, 60 min); CTRL > both; AA (180 min): KA > both**; AA (120 min): PEA2/KA vs. KA vs. CTRL, NS; **PGE2 (60 min): KA > CTRL; PGE2 (120, 180 min): KA > both**; PGE2 (20 min): PEA2/KA vs. KA vs. CTRL, NS; **PGD2 (120, 180 min): CTRL < both**; PGD2 (20, 60 min): PEA2/KA vs. KA vs. CTRL, NS; **AEA (20, 60, 120, 180 min): PEA/KA < URB597/KA, PEA + URB597/KA; 2-AG (20, 60, 120, 180 min): PEA/KA > both; PEA (20, 60, 120, 180 min): PEA + URB597/KA > both; AA (20 min): PEA/KA < PEA + URB597/KA; AA (60, 120 min): PEA/KA < both**; AA (180 min): PEA/KA vs. both, NS; **AEA (20, 60, 120, 180 min): PEA/KA < URB937/KA, PEA + URB937/KA; 2-AG (20, 180 min): PEA/KA > both; 2-AG (120 min): PEA/KA > PEA + URB937/KA**; 2-AG (60 min): PEA/KA vs. both, NS; **PEA (20, 120 min): PEA/KA < PEA+ URB937/KA; PEA (60, 180 min): PEA/KA < both**; AA and PGD2 (20, 60, 120, 180 min): PEA/KA vs. both, NS; **PGE2 (20, 60 min): PEA/KA vs. both, NS; PGE2 (120, 180 min): PEA/KA < PEA + URB937/KA**. 2. (a) **NeuN staining: KA**, ↓; PEA2/KA vs. saline, NS; (b) **FJC staining (5 days post KA): PEA2/KA < KA**; (c) Silver staining: KA, ↑↑; PEA2/KA ↑; saline, ─						

PEA, palmitoylethanolamide; ECBs, endocannabinoids; ASMs, antiseizure medications; MES, maximal electroshock seizures; VHI, vehicle; min, minutes; h, hour/hours; mg/kg, milligrams per kilogram; AEA, anandamide; PA, palmitic acid; PAA, palmitamide; HXD, hexadecanol; 16HPA, 16-hydroxypalmitic acid; ED50, median effective dose; CIS; chemically induced seizures; PTZ, pentylenetetrazol; MPA, 3-mercaptopropionic acid; BC, bicuculline; STR, strychnine; PIC, picrotoxin; NMA, N-methyl-D,L-aspartate; KS, kindled seizures; PPAR-α, peroxisome proliferator-activated receptor alpha; AEs, acylethanolamines; icv, intracerebroventricular injection; ip, intraperitoneal injection; SR, SR141716 (CB_1_ receptor antagonist); GW, GW6471 (PPAR-α antagonist); EEG, electroencephalogram; LC/APCI/MS, liquid chromatography-atmospheric pressure chemical ionization-mass spectrometry; CB1, Cannabinoid receptor type 1; P14, postnatal day 14; P56–70, postnatal day 56–70; KA, kainic acid; TLC, thin layer chromatography; LC-ESI-MS, liquid chromatography-electrospray ionization-mass spectrometry; PSs, population spikes; DMSO, Dimethyl sulfoxide; AM630, CB_2_ receptor antagonist; AM251, CB_1_ receptor antagonist; CB2, Cannabinoid receptor type 2; NIDA, NIDA-41020 (CB_1_ cannabinoid receptor antagonist); ACEA, arachidonyl-2’-chloroethylamide; PLs, phospholipids; eiCs, eicosanoids; LC-MRM, Liquid Chromatography-multiple Reaction Monitoring Mass Spectrometry; PEA2, subchronic PEA; CTRL1, Vehicle 2 injection; CTRL2, Vehicle 1 + 2 injection; URB597, selective inhibitor of fatty acid amide hydrolase (FAAH); URB937, peripheral inhibitor of FAAH; vs., versus; NS, not significant; TD50, median toxic dose; PI, protective index; AD, afterdischarge; SWDs, spike-wave discharges; µg, micrograms; µL, microliters; WAG/Rij, Wistar Albino Glaxo from Rijswijk (rats); ACI, August Copenhagen Irish (rats); NAPE-PLD, N-acyl phosphatidylethanolamine-specific phospholipase D; CBR, cannabinoid receptor; FAAH, fatty acid amide hydrolase; MAGL, Monoacylglycerol lipase; DAGL, Diacylglycerol lipase; JZL, JZL184 (irreversible inhibitor for MAGL); LP, latency period; S5D, stage 5 duration; S5L(-1), stage 5 invers onset; SS, seizure stages; CBZ, carbamazepine; DZP, diazepam; FBM, felbamate; GPT, gabapentin; LTG, lamotrigine, OXC, oxcarbazepine; PB, phenovbarbital; TPM, topiramate; VPA, valproate; LEV, levetiracetam; PHT, phenytoin; cCTX, cerebral cortex; PA16:0_18:1, 1-hexadecanoyl-2-(9Z-octadecenoyl)-sn-glycero-3-phosphate; SM d18:1/18:0, sphingomyelin d18:1/18:0; PE 16:0_18:1, phosphatidylethanolamine 16:0/18:1; PE 20:2_20:4, phosphatidylethanolamine 20:2_20:4; 12(S)-HETE, hydroxyeicosatetraenoic acid-d8; 15(S)-HETE, 15(S)- hydroxyeicosatetraenoic acid; CER, cerebellum; OEA, oleoyl ethanolamide; LPC20:4, lysophosphatidylcholine 20:4; PC 18:2_20:4, phosphatidylcholine 18:2_20:4; THL, thalamus; PE 20:0_20:4, phosphatidylethanolamine 20:0_20:4; C16:0, palmitic acid; PS 16:0_18:1, phosphatidylserine 16:0_18:1; HYP, hypothalamus; PG 18:1_20:4, phosphatidylglycerol 18:1_20:4; PE 18:0_20:4, phosphatidylethanolamine 18:0_20:4; PE 20:2_20:4, phosphatidylethanolamine 20:2_20:4; LPC 18:0, lysophosphatidylcholine 18:0; PC 16:0_18:1, phosphatidylcholine 16:0_18:1; PG 16:0_18:1, phosphatidylglycerol 16:0_18:1; PI 16:0_18:1, phosphatidylinositol 16:0_18:1; PC 18:0_20:4, phosphatidylcholine 18:0_20:4; PG 16:1_20:4, phosphatidylglycerol 16:1_20:4; PE 18:2_20:4, phosphatidylethanolamine 18:2_20:4; LPA 16:0, lysophosphatidic acid 16:0; LPA20:4, lysophosphatidic acid 20:4; C18:1, oleic acid; PGD2, prostaglandin D2; HC, hippocampus; PGF2α, prostaglandin F2α; STR, striatum; C20:4, arachidic acid; PC 18.2_20:4, phosphatidylcholine 18.2_20:4; AA, arachidonic acid; PGE2, prostaglandin E2; 2-AG, 2-Arachidonoylglycerol; NeuN, neuronal nuclear protein; FJC, Fluoro Jade C. Bold font emphasizes statistically significant results.

**Table 2:** Methodological quality of animal studies investigating palmitoylethanolamide and its correlations to epilepsy and acute seizures.

Study (Country)	Study Design	Defined Study Population	Age	Gender	PEA Measure	Adequate PEA Evaluation	Control Group	Statistical Analyses	Funding or Sponsorship
Lambert et al. (2001) (Belgium)	√ Analytic, observational, interventional	√ OF1 mice	X	√ Male	√ 1. MES test: 50, 100 mg/kg (ip); Time course calculation: 25 mg/kg (ip); ED50 calculation: 0.5–50 mg/kg (ip); CIS test: 25 mg/kg (ip) 2. Rotarod test: up to 250 mg/kg (ip)	√ 1. (a) MES test: double assessment (30 min, 4 h); (b) Time course calculation, ED50, CIS test: single administration. 2. Rotarod test: multiple administrations	√ 1. MES test: VHI (30 min/4 h); CIS test: VHI, PHT 2. Ameltolide, PHT, VPA, PB, CBZ	√ Fisher’s exact test	X
Sheerin et al. (2004) (Canada)	√ Analytic, observational, interventional	√ Long–Evans rats	X	√ Male	√ KS test: 1, 10, 100 mg/kg (ip); CIS test: 40 mg/kg (ip)	√ Single administration, 2 h before each kindling session	√ KS test: [VHI, PEA(1), PEA(10), PEA(100)] baseline; CIS test: VHI	√ ANOVA; Fisher’s exact test; *t*-test	√
Citraro et al. (2013) (Italy)	√ Analytic, observational, interventional	√ WAG/Rij, Wistar, ACI rats	√ 1 month; 6–7 months	√ Male	√ 1. 0.5, 1, 3, and 10 µg/2 µL (icv); 10, 20, 40, and 60 mg/kg (ip); 20 and 40 mg/kg (ip post SR); 3 µg/2 µL (icv post GW) 2. Brain tissue levels	√ 1. (a) Single administration after 1 h baseline EEG recording; (b) single administration after 1 h baseline EEG recording and 30 min after SR or GW administration; 2. Double assessment (2 and 6 months)	√ 1. VHI (icv/ip) 2. Wistar, ACI rats	√ ANOVA; Tukey’s post-hoc test	√/X
Fezza et al. (2014) (Italy)	√ Analytic, observational	√ Wistar rats	√ P14 and P56–70	√ Male and female	√ Brain tissue levels	√ Single assessment	√ Saline	√ ANOVA; *t*-test; Mann–Whitney U test	√
Aghaei et al. (2015) (Iran)	√ Analytic, observational, interventional	√ Wistar rats	√ 8–10 weeks	√ Male	√ 1, 2.5, 5, 10, 25 µg/kg (icv)	√ Single administration	√ VHI	√ ANOVA; Mann–Whitney U test; Kruskal–Wallis test; Tukey’s test	√
Citraro et al. (2016) (Italy)	√ Analytic, observational, interventional	√ DBA/2 mice	√ 22–26 days or 48–56 days	√ Male	√ 5–40 mg/kg (ip)	√ Single administration 30, 60, 90, or 120 min before auditory stimulation	√ VHI, ASMs + VHI	√ ANOVA; Fisher’s exact test; Dunnett’s test; χ2-test; *t*-test	√
Lerner et al. (2017) (Germany)	√ Analytic, observational	√ C57BL/6N mice	√ 80–100 days	√ Male	√ Brain tissue, peripheral tissue, plasma levels	√ Single assessment after 1 h KA-injection	√ Saline	√ ANOVA; Shapiro–Wilk test; Kolmogorow–Smirnow test; *t*-test	√
Post et al. (2018) (Germany)	√ Analytic, observational, interventional	√ C57BL/6N mice	√ 8–10 weeks	√ Male	√ 1. 40 mg/kg (ip); 2. Brain tissue levels; plasma levels	√ 1. (a) Single administration (acute treatment, 30 min prior to KA); (b) double administration (subchronic treatment, 7 h and 30 min prior to KA); 2. Multiple assessment	√ 1. KA, CTRL1, CTRL2; 2. saline	√ ANOVA; Greenhouse–Gasser correction, Bonferroni’s post-hoc analysis for multiple comparisons	√

MES, maximal electroshock seizures; mg/kg, milligrams per kilogram; ip, intraperitoneal injection; ED50, median effective dose; min, minutes; h, hours; VHI, vehicle; PHT, phenytoin; VPA, valproate; PB, phenobarbital; CBZ, carbamazepine; KS, kindled seizures; ANOVA, Analysis of Variance; WAG/Rij, Wistar Albino Glaxo from Rijswijk (rats); ACI, August Copenhagen Irish (rats); µg, micrograms; µL, microliters; icv, intracerebroventricular injection; SR, SR141716 (CB_1_ receptor antagonist); GW, GW6471 (PPAR-α antagonist); EEG, electroencephalogram; P14, postnatal day 14; P56–70, postnatal day 56–70; ASMs, antiseizure drugs; KA, kainic acid; CTRL1, Vehicle 2 injection; CTRL2, Vehicle 1 + 2 injection.

## Data Availability

Not applicable.
